# “[He] can be supportive, but at times I feel he is ashamed of me”: Understanding the relationship between parental support and quality of life amongst trans and gender diverse youth in the UK

**DOI:** 10.1080/26895269.2023.2286269

**Published:** 2024-02-05

**Authors:** Abby Barras, Bethany A. Jones

**Affiliations:** a School of Humanities and Social Science, University of Brighton, Brighton, UK; b Psychology, Nottingham Trent University, Nottingham, UK

**Keywords:** Gender-diverse, parental support, quality of life, transgender, youth

## Abstract

**Background:**

Trans and gender diverse (TGD) youth often report poor relations with their parents and perceive these to be core to the mental health difficulties they experience. One aspect of psychological wellbeing that has not been well explored in relation to parental support is Quality of Life (QoL).

**Aim:**

To test the association between perceived parental support and QoL and, understand from the young person’s perspective how parental support contributes to QoL.

**Method:**

To address these aims a multi-methods design was used and 140 TGD youth aged 11–19 years old from the UK took part in an online survey in 2020. Validated measures of parental support and QoL were used in conjunction with open-ended survey questions about experiences of parental support.

**Findings:**

As expected, we found a significant and positive association between parental support and QoL. Two themes were found in the qualitative data: (1) Parental support is not black or white, (2) Knowledge is a catalyst for affirmative parental support.

**Conclusions:**

Our findings demonstrate the positive implications of affirmative family support on QoL but at the same time highlight how parental relations can be complex and frequently conditional. Organizations supporting young TGD people (e.g. those working in education, healthcare) should prepare young people for the complexity of family relationships. Knowledge and awareness were felt to be an important tool in increasing the likelihood of parental support, but affirmative and evidence-based support needs to be made more readily available.

## Introduction

It is well established that trans and gender diverse (TGD) youth are vulnerable to poor mental health and wellbeing (e.g. Drabish & Theeke, 2022; Jones et al., 2019, 2023). Within the field, it is widely acknowledged that the high prevalence of poor mental health among TGD youth has a social cause and therefore can be attributed to unwelcoming and harmful experiences often encountered (Brooks, 1981; Hendricks & Testa, 2012; Meyer, 2003; Riggs & Treharne, 2017). Social stressors can be both interpersonal (e.g. peer rejection, bullying) and structural (e.g. inability to obtain legal recognition of gender identity, barriers in accessing gender affirming care; e.g. Testa et al., 2015). One aspect which is commonly understood to have a strong influence on the mental health and wellbeing of young TGD people is parental support (Gaspar et al., 2022; Hendricks & Testa, 2012).

Parental support that affirms a young person’s gender identity (e.g. using correct pronouns) is associated with an increased likelihood of living as one’s affirmed gender, fewer mental health symptoms, reduction of suicidal ideation, and increased help-seeking (Russell et al., 2018; Samrock et al., 2021; Tan et al., 2021; Weinhardt et al., 2019). However, supportive parental bonds can be hard to come by for TGD youth (Fuller & Riggs, 2018; Pullen Sansfaçon et al., 2020). Research with parents of TGD youth has cited a range of barriers to providing support which has included their beliefs and attitudes about gender (e.g. essentialist beliefs), their emotions (e.g. fear over the safety of their young person, grieving a future dream of being a grandparent), perceiving a lack of social support (e.g. not knowing other families with TGD youth, anticipating negative evaluation from wider family) and, a lack of knowledge about gender identity and TGD challenges (e.g. Matsuno et al., 2022; Morgan, Wells, et al., 2022; Pullen Sansfaçon et al., 2020; Wagner & Armstrong, 2020). There is limited research to date that has triangulated these perspectives with those of TGD youth which limits the effectiveness of parental support initiatives.

Unsupportive parental relationships have been associated with psychological distress in TGD youth, including suicidality (Price & Green, 2023) and unhealthy coping behaviors (e.g. alcohol and drug misuse; Winter et al., 2016). However, one aspect of psychological wellbeing that has not been well explored in relation to parental support is Quality of Life (QoL) (Engel et al., 2023). QoL does not have a generally accepted definition (Tvaronavičienė et al., 2022), but can be best understood as:

A broad ranging concept incorporating in a complex way the persons’ physical health, psychological state, level of independence, social relationships, personal beliefs and their relationships to salient features of the environment (World Health Organisation [WHO], 2012, p. 11.)

TGD youth have been found to report poorer QoL when compared to cisgender youth, even those who have health conditions known to be associated with poorer QoL (e.g. poor mental health, cancer, asthma; Engel et al., 2023; O’Bryan et al., 2020; Röder et al., 2018; Simons et al., 2013; Zou et al., 2018). However, a recent systematic review highlights how there are no studies that have explored the social determinants of QoL (e.g. parental support) among TGD youth.

## The current study

Research conducted by Engel et al. (2023) has revealed that the QoL among TGD youth is notably lower in comparison to their cisgender peers. However, there remains an unaddressed question regarding the link between QoL and social factors, particularly parental support. The experiences of TGD youth have been largely omitted from the current understanding. Most research has predominantly focused on the perspectives of parents when investigating the barriers and enablers to support. An exception to this trend is the study by Morgan, Raab et al. (2022), which explicitly delved into the perceptions of young TGD individuals regarding the obstacles and catalysts in parental support. One of the most instrumental barriers identified was a lack of credible knowledge about gender identity available to their parents. However, this study was conducted in Australia and whilst enlightening, its findings are not reflective of the current UK climate. Globally, TGD people’s rights differ and consequently the support available to young TGD people and their families is subject to change. To improve the parental support available, further research is needed cross-culturally to ensure initiatives are sensitive to the social and cultural climate of TGD youth rights. To our knowledge, no research has explored the perceptions of parental support among youth residing within the UK.

The objective of this study is to bridge existing gaps in the current body of evidence by examining the relationship between parental support and the QoL of TGD youth in the UK. Additionally, this research aims to gain insights into the obstacles hindering and the factors fostering parental support as perceived by the young individuals themselves. Based on previous research (e.g. Hendricks & Testa, 2012), it was hypothesized that parental support would positively correlate with QoL. Following on from this we used open-ended survey data to explore young TGD people’s perceptions of barriers to and, facilitators of affirming parental support.

## Method

### Design, participants and recruitment

To address the research aims a multi-method design encompassing both open and closed survey questions was chosen. This enabled us to understand, in more depth, why TGD youth felt their parents to be (un)supportive and thus be able to make more specific recommendations. Participants were recruited through Mermaids, a UK charity who support TGD youth and their families. Adverts were placed on Mermaids secure online forums and social media platforms (e.g. Twitter) using written posts and videos that advertise the project. Advertising in this way ensured reach to a diverse participant sample. To take part, participants were asked to self-identify as TGD and be between 11 and 19 years old. Nottingham Trent University ethics board provided ethical clearance for this research.

### Measures and procedures

Questionnaires were distributed *via* Survey Monkey, an online, cloud-based software package. The survey ran in the winter of 2020, and participants were asked to complete demographic questions relating to themselves and their parents and then asked to complete the following validated measures:

### Quantitative measures

*Parental Attitudes of Gender Expansiveness for Youth (PAGES-Y) (Hidalgo et al., 2017)* questionnaire was used to assess participants’ perceived parental support (e.g. “*My parent(s) are supportive of my gender transition*”). Participants were asked to complete the measure up to two times, for different parents/carers. Participants were able to define who these parental figures were (see Table 1). The tool includes 14 items and was scored on a 5-point Likert scale (1 = strongly disagree − 5= strongly agree). The total score was calculated using the mean and, a higher score represented greater perceived parental support. The measure has previously been found to be reliable (*α* = .94; Hidalgo et al., 2017) and this was replicated in the current sample (*α* = .96 for parent 1 and *α* = .96 for parent 2).

**Table 1.** Demographic variables for sample (*N* = 140).

Variable		*N* (%)
Gender	Male	68 (48.6)
	Female	36 (25.7)
	Nonbinary	29 (20.7)
	Other	6 (4.3)
	Prefer not to say	1 (0.7)
Ethnicity	White British	117 (83.6)
	Asian Indian	3 (2.1)
	Black Caribbean	1 (.7)
	White Irish	6 (4.3)
	Mixed	2 (1.4)
	Missing	11 (7.9)
Education & Employment	School	50 (35.7)
	Sixth form	31 (22.1)
	College	33 (23.6)
	University	10 (7.1)
	Full-time work	1 (.7)
	PT work	4 (2.8)
	Other	11 (7.9)
Parent 1	Mother	98 (70)
	Father	25 (17.9)
	Both	1 (.7)
	Stepmother	1 (.7)
	Grandmother	1 (.7)
	Adopted parent	1 (.7)
	Missing	13 (9.3)
Living with parent 1	Yes	113 (80.7)
	No	7 (5)
	Sometimes	7 (5)
	Missing	13 (9.3)
Parent 2	Mother	23 (16.4)
	Father	74 (52.9)
	Step farther	4 (2.9)
	Stepmother	1 (.7)
	Mothers’ partner	1 (.7)
	adopted parent	1 (.7)
	Uncle	2 (1.4)
	Missing	34 (24.3)
Living with parent 2	Yes	77 (55)
	No	16 (11.4)
	Sometimes	12 (8.6)
	Missing	35 (25)

*The Good Childhood Index (*The Children’s Society, 2020*)* was used to assess QoL. The tool comprises of two sections, the first that includes five questions that assess overall life satisfaction (e.g. “*I have a good life”*) and a separate 11 items that ask about happiness in the context of different life domains (e.g. School, housing, appearance). Only two items from the latter section were used in the current study, specifically the item about satisfaction with family relationships (i.e. “*How happy are you with your relationships with your family?”)* and overall happiness (i.e. “*How happy are you with your life as a whole?”*). As single items were used, reliability statistics could not be calculated. Items are scored on a 10-point Likert scale where “0” is not happy at all and “10” is completely happy. Scores between 0-4 indicate very poor QoL.

### Qualitative questions

Alongside these validated measures, participants were asked to complete nine open-ended survey questions about their experiences of parental support. This included asking about concealment of gender identity with parents (*“Have you told Parent 1/2 about your gender identity”*), perceived parental support (*“Overall, when it comes to your gender identity, do you feel supported by Parent 1/2”*), parental reactions to gender (*“Do you feel that Parent 1 and Parent 2 had similar or different reactions towards your gender identity?”*), gender identity knowledge *(“How much does Parent 1/2 know about trans identities”*) and, LGBTQ + community contact (*“Other than you, does Parent 1/2 know any LGBTQ + people”*). These question topics were developed in collaboration with a former Mermaids employee who self-identifies as transgender. This allowed for the necessary sense checking and ensured questions were representative of the TGD community (Vincent, 2018).

### Quantitative analysis

IBM SPSS version 28 (IBM Corp, 2021) was used to conduct all quantitative analysis. Descriptive statistics were calculated for all study variables. Normality testing demonstrated that the data were non-normally distributed and therefore, where available, non-parametric tests were conducted. Spearman’s correlations were used to explore associations between all study variables.

### Qualitative analysis

The analysis of the qualitative data was guided by Braun and Clarke (2006; 2022) work on thematic analysis. First familiarization of the data took place by reading and re-reading the open responses gathered *via* the survey. Initial ideas about patterns and meaning within the data were noted. Following this, the data were coded inductively, which involved broadly identifying relevant words, phrases and sentences. This process was done manually, using colored highlighters, flagging codes in different colors. Codes were reviewed a second time by both authors allowing for a re-checking of the labeling being carried out, and to prevent occurrences of different terms being used to describe the same instances (Bryman, 2008). At this stage, data not relevant to the study were discarded as agreed by the authors.

Next, themes were developed by clustering codes that were felt to have shared meaning and were organized thematically by the first author. The first author focused on identifying themes which were “internally coherent, consistent and distinctive” (Braun & Clarke, 2006, p. 87), which is both systematic and flexible in helping to answer the research question and is also in keeping with an interpretivist ontology. Extracts were then collated under each theme and then both authors then met to discuss the thematic structure. Several iterations of re-organising the thematic structure were undergone until it was finalized to ensure the overall narrative and connection between themes was clear. The analysis resulted in two themes (with three subthemes) being developed (see results section).

The two authors of this study are cisgender females, which means they do not have direct personal experiences related to the subject matter. Nevertheless, their substantial research experience and deep engagement with the existing literature in this field have likely shaped their interpretations. To maintain transparency and self-awareness during the data analysis phase, the authors maintained a reflexive journal. This journal allowed them to reflect on how their own values and positions might influence their understanding and interpretation of the research findings.

## Results

### Quantitative results

#### Demographics

The mean age of the sample was *M* = 15.91, *SD* = 1.9. Prior to data cleaning, the sample comprised of 153 young people who met the inclusion criteria. The final analyzed data set consisted of *N* = 140. Thirteen people were removed as their responses were incomplete and therefore not analyzable. Most indicated that their primary parental figure was their mother whom they lived with. Most identified their father as the secondary parental figure, and they most commonly lived in the same home (see Table 1).

### Quantitative correlations

Affirming support from parent 1 (*M* = 3.59 *SD* = 1.07) was positively and significantly correlated with QoL overall (*r* = .49, *p* < .001; *M* = 4.88; *SD* = 2.42) and satisfaction with family relationships (*r* = .67, *p* < .001; *M* = 5.61; *SD* = 2.54) and this was also the case with affirming support from parent 2 (*M* = 3.46; *SD* = 1.03) for overall life satisfaction (*r* = .43, *p* < .001) and satisfaction with family relationships (*r* = .51, *p* < .001).

### Qualitative findings

In this study two main themes and three subthemes relating to perceived family support were identified in the analysis (see Table 2).

**Table 2.** Themes and subthemes identified in analysis.

Theme	Subtheme
1. Parental support is not black or white	1a. Actions speak louder than words
	1b. Conditional parental support
	1c. Unintentional mistakes
2. Knowledge is a catalyst for affirmative parental support	

## Theme 1: parental support is not black or white

Many young people described how support from their parents could feel conflicting and confusing, and most importantly, inconsistent. For example, one young person describes how:

My dad is supportive in that he supports me getting HRT, but he has been extremely resistant to getting my name changed and is by far the slowest person in my life regarding using my new name and pronouns. He also feels like things that cause me dysphoria “shouldn’t really bother you [me] that much”. He’s only gone to one support session for parents, and when I try to inform him of my dysphoria he seems to not understand and gets defensive.

When parental support was conflicting it often left participants feeling unworthy and isolated:

He tries his best and can be supportive, but at times I feel he is ashamed of me and struggles to see me for myself and struggles to talk about me to anyone outside of the family.

Inevitably, these experiences took their toll on an individual’s QoL, whereby in the context of the culture and value systems inherent in their family, they perceived their own position to be diminished. The following subthemes expand on how experiences of conflicting support manifested for young people in this study, and how they managed their responses to it.

## Subtheme 1a) actions speak louder than words

Participants described how their parents had verbalized that they were supportive of their gender identity but did not always actively display support for their young person. For example:

While he appears on the surface to support me, he refuses to substantiate that with facilitating any actual steps towards my transition, no matter how much I plead.

This meant young people felt that their experiences were ignored and not taken seriously by their families. Below, one participant discusses this experience. Their parent is aware of their gender identity but does not acknowledge or validate it and therefore is not able to provide their young person with much-needed emotional support:

I don’t feel supported so much as just kind of accepted, I don’t feel that I can talk about ‘trans things’ with her and she still holds some rather dated and stereotypical gender views although doesn’t actively voice them. I don’t think she really understands being non-binary, so I haven’t discussed it.

For this young person and many others in this study, the support they receive is insufficient due to a blanket lack of acknowledgement (supportive or otherwise) about their gender identity from their parent. In addition, even if they could broach the topic of their gender identity, their parent holds views which would be unlikely to lead to a supportive, gender affirming conversation. This indicates non-binary youth are less likely to open up the discussion further, and as other research has found, may find themselves feeling less supported/visible than binary trans youth (Weinhardt et al., 2019).

## Subtheme 1b) Conditional parental support

Many young people described conditions that were placed on their gender identity by their parents. It was common for young TGD people in this study to feel as if their gender identity was influenced and controlled by their parents. For example, some explained how their parents were happy for them to live authentically at home, but not outside of the home especially around extended family (*“They won’t let me tell other family members but are relaxed at home”).* Conditional support was also experienced in the way a parent would offer partial support (“*My dad is supportive in that he supports me getting HRT, but he has been extremely resistant to getting my name changed and is by far the slowest person in my life regarding using my new name and pronouns”).* This left young people feeling as if their gender identity and expression was something which could be controlled by their parent(s):

The second time I brought up the topic I was trans I was asking to be called by my chosen name, he proceeded to call me "daft". He won’t call me by my chosen name because "I chose your name for you".

Parents also gave off the impression that their gender identity and expression was something they would grow out of and therefore age was a common condition prescribed to transition:

On the one hand, she has told me she accepts me, and we’ve had some talks about gender, but she also doesn’t want me to transition until I am older (early mid 20s).

In some examples a parent would simply refuse to believe their child was TGD (“*He just doesn’t believe nonbinary exists and he doesn’t take my concerns seriously*”), and use their position to avoid talking about the topic, and by extension, flatly denying any support (“*She believes that [being trans] is a choice, being faked for attention [and] people who are trans are lesser people than anyone else or worthy of respect*”). This suggests parents perceived gender identity as a “choice” and something that could be easily changed with no psychological consequences.

## Sub-theme 1c) unintentional mistakes

Many young people reflected on how their parents would make mistakes (e.g. misgendering, deadnaming) when it came to demonstrating gender-affirming support. While this was evidently distressing, young people were able to accept that this was part of the journey that their parents were on. For example, one young person said:

Although she struggled to understand the concept at first, she has always been willing to learn. Sometimes she still gets my pronouns wrong, which can irritate me, but she has no malicious intent and I know she does try. Overall, she has done her best to support me despite messing up on some occasions such as making some assumptions, misgendering me, and coming out for me when I would have rather she asked for permission first.

These mistakes were inconsistent, and parents would often show affirmative and non-affirmative support simultaneously. This helped young people reason that the latter was not an indicator of a lack of overall support. For example:

She’s been misgendering me for years, though, she writes my name as preferred. When we got Netflix this year, for example, she made my account have my chosen name without me even telling her to and is very clearly not against me being trans so that’s why I say partly [supportive]. I think she’s just really making common mistakes I should expect of parents her age and in actuality, she’s very supportive!

Mistakes were often described as “common” or to be expected from their parents. Many young TGD people in this study were sympathetic of these mistakes which enabled them to maintain a relationship with their parents. Several young people reflected on how they did not feel their parent’s mistakes were intentional or malicious. Often young people in this study reasoned that although hurtful, these mistakes came from a place of misunderstanding and lack of knowledge surrounding gender identity:

Parent 2 has always tried to understand my identity but can say insensitive things at times down to lack of understanding. He often gets my pronouns wrong 11 months down the line, but I know he has no malicious intentions. Overall, I feel supported, but less so than by Parent 1.

Overall, non-affirming parental support, when experienced simultaneously with positive, gender affirming support, was not felt to be intentional. Instead, these ‘mistakes’ were perceived as a result of misinformation and a lack of knowledge about gender. The next and final theme will explore the role that knowledge played in affirming support available from parents. Such parents were able to demonstrate how they were able to bring gender diversity into their consciousness as a reality, not as a transient phase, and one they wish to support (Riggs, 2019). Knowing this and feeling as if their parent was at least trying to be supportive was sufficient for participants to not hold these mistakes against their parents.

The findings here reflect a mix of support young people received from their parents. We suggest they offer a shading in of the grey space between the barrier/faciliatory binary tropes of either unconditional support/no-support. Like previous research (Fuller & Riggs, 2018; Morgan, Wells, et al., 2022; Morgan, Raab, et al., 2022; Roe, 2017), we noticed examples ranging from no support and estrangement (“*He was not accepting when I asked to go by a different name and said I wasn’t his child now”*), to unconditional acceptance (“*She has been very very supportive of me since day one. She is one of my biggest inspirations and has never judged me for who I am. She knows that this is me and no one can change that*”). Although young people in this study evidently felt that “some” support was better than none, our findings indicate that conditional support was a strong yet subtle factor when considering perceived parental support, and its impact on QoL.

## Theme 2: knowledge is a catalyst for affirmative parental support

Young people in this study reported how negative attitudes and limited family support were linked to a lack of education and knowledge about gender issues. Some young people had to navigate this carefully and used this understanding of their parents’ knowledge and attitudes to decide whether they needed to conceal their gender identity or felt able to live authentically in their gender identity. For example, one participant spoke about their parents’ negative attitudes toward TGD people, and how they are “*forced to hide out of fear of getting my binders thrown away,*” preferring not to come out and risk this happening.

The media also played a part in parental knowledge formation and was sometimes a site of tension. Many young people reflected on how they felt the media had negatively influenced their parent’s attitudes toward TGD gender identities. For example, one participant said:

Like my mum, dad had only really heard about trans people in the media so had lots of preconceived ideas about what a trans person’s experiences may be like, and since I didn’t fit his expectations, [he] was doubtful of my gender identity initially.

For some young people, this was problematic as parents often viewed this form of knowledge as superior to all other forms of knowledge. This meant that young people’s voices were lost, and their feelings and unique experiences were not validated against the backdrop of negative media narratives. It was evident across the dataset that media narratives were perceived as harmful by young people and prevented them from accessing support from their parents and families. However, one young person described how they were able to overcome the influence these narratives had on parental attitudes with patience and perseverance:

[She] claimed to know more than she really did, picking and choosing the facts from news sources pushing certain narratives, when I came out. So, it took lots of patience but now she is much more educated. Since I’ve made it clear to her that I know what I’m talking about, she respects my identity and is willing to learn from me first-hand.

Alongside patients, young TGD people in this study also needed to be assertive to facilitate a change in parental attitudes which many TGD youth may find challenging given the hierarchy nature of family relations. Despite this, it was clear that young people, although often dissatisfied with perceived parental support, accepted it was often a work-in-progress for all involved, which could result in support that was validating and affirmative. This was even possible in cases where parents were described as having very traditional or strong views about gender which might have been perceived as being too powerful for education to be effective:

He had a very traditional background was a little small minded he wasn’t particularly understanding or caring towards LGBT + people but has learned to see the world from a different perspective and is starting to be a lot more supportive.

It was evident that education was transformative for parents as the young people in this study felt that their parents often began to see the world through a new, more accepting lens once educated about gender diversity. They did discuss the “significant” effort that was required from their parents to undergo this process:

Since I came out to Parent 2, he has made a significant effort to learn about trans identities by looking online for more information. It was online where he found out about [support organisation] who aided him in his understanding of trans identities, further helping him in accepting me as non-binary.

Participants alluded to their parents needing to be in a place of readiness for education to be truly effective. In some cases, knowledge was a catalyst for improved support. Educated parents were able to further support their young person by sharing awareness more broadly:

Once I came out to Parent 1, her knowledge of trans identities improved to the point that she is happy educating people around her who have less/no knowledge about trans identities.

These findings underscore the crucial role that the lack of knowledge plays in shaping parental attitudes toward TGD youth. It is evident that many parents derive their initial understanding of gender diversity from mainstream media, which, unfortunately, is often unreliable. Some TGD people in this study felt able to educate their parents although this often demanded a substantial amount of patience and persistence on their part. However, educating parents often improve family relations and subsequently the support available to them, even in cases where parents held firmly entrenched traditional beliefs rooted in gender essentialism. This demonstrates the transformative potential of informed and empathetic communication, ultimately fostering understanding, acceptance, and support for TGD youth within their families.

## Discussion

The purpose of this study was to better understand what TGD young people’s experiences were of perceived parental support, and how this might impact—positively or negatively—on their QoL. As expected, the quantitative data demonstrated a positive relationship between perceived parental support and QoL, whilst the qualitative data expanded on these findings to better understand how young TGD experience support from their parents or carers. Specifically, these findings demonstrated that consistent affirmative family support was hard to come by.

### Perceived support vs. real-life support

As shown in the quantitative findings, affirming support from parent 1 and parent 2 was positively and significantly correlated with QoL, as in the existing research (e.g. Engel et al., 2023), and this was reflected in the qualitative findings, with young people reporting feelings of happiness, security and empowerment when their parent(s) demonstrated solidarity. However, we also found that parental support was not always black and white. Rather, it was conditional, inconsistent and not always obvious. Similar to Andrzejewski et al. (2021), we found parental support to span several different domains (emotional, instrumental, appraisal and informational), but we also found support sometimes shifted between these domains, and may be simultaneously supportive and unsupportive (e.g. gender identity was affirmed inside the home, but not outside of it). In this way, young people found themselves having to choose which aspects of support were of most value to them. This included, for instance, having a parent support the use of gender-affirming hormones, whilst simultaneously deadnaming them. This brings an important dimension to this conversation, whereby QoL is reliant on full support, not partial, and like Morgan, Raab, et al. (2022, p. 48) we acknowledge how such a shortcoming of support can exacerbate minority stress “which can impede parental ability to support their child’s gender identity in a cisnormative world.” These findings tell us what TGD youth perceive to be supportive and therefore is useful for organizations offering support and advice to families with TGD youth.

### Patience and perseverance

For the TGD youth in our study, they recognized that with patience and perseverance, mistakes, such as deadnaming and misgendering, could be moved past and were part of the evolving relationship between themselves and their parent. As Morgan, Raab, et al. (2022) note (drawing on Travers et al. (2012)), having a “somewhat supportive” parent did not result in a more positive effect on the young person than if parents were in the “not at all supportive” category (Travers et al., 2012). Reflecting on the WHO’s definition of QoL (2012) (other than when it is unrelated to physical health), feeling fully supported by their parent(s) is key to TGD youth achieving this. We have seen in the data the positive effect this support has on other salient aspects of their life, such as coping mechanisms, and the opposite, such as feeling unworthy, when support is not forthcoming. Some of our participants recognized that their parent(s) was accepting and supporting but still made mistakes, highlighting their willingness to be responsible in effecting harmonious change within the family unit (Pullen Sansfaçon et al., 2015), and indicates they feel secure enough to do so. Family harmony matters for many TGD youth, and many were able to eventually recognize the complexity of relationships. However, this conflicting support can initially be confusing for the young person and difficult to navigate. Support organization should work to prepare young TGD people for what are often very complex and challenge relationships.

### Learning together

In some cases, parents drew upon inaccurate knowledge produced in the mainstream media, and in this study TGD youth were aware of the influence hostile media and misleading public narratives can have on the support they receive from their parent(s), as similarly identified in previous research (Morgan, Raab, et al., 2022; Pham et al., 2020). Many TGD youth look to TGD elders as role models, and if their parent(s) is an ally, this can offer them comfort and reassurance that they will be accepting of their own TGD identity as something not new, but an established reality. The experiences of the young people in our study show how they believed knowledge could sometimes be reimagined and reframed as an opportunity for a parent(s) to learn from their young person, explore new ground together, and that reactions to knowledge could change over time (Morgan, Raab, et al., 2022). In light of these findings, organizations offering support to young TGD people and their families should also ensure information and resources available online are easily available, evidence-based and affirmative.

### Limitations and future research directions

Two limitations are worth considering with the interpretations of our findings. Firstly, we did not closely scrutinize sociodemographic factors such as age, education, race/ethnicity, religion, disability or socio-economic status. The participants in our study were overwhelmingly White British. As such our sample is not wholly representative of the TGD population. This is an area in need of urgent consideration because, as Kuvalanka and Munroe (2020, p. 598) write

Although research in this area has increased in the past decade, more studies—especially those involving parents of color, families from lower socioeconomic backgrounds, and caregivers of children with nonbinary gender identities—are needed to better understand what practices best support the well-being of trans youth.

Secondly, as we have found, parental support is multi-layered and not black and white. One area for further study would be examining support from other family members, such as siblings, many of whom play both supportive and unsupportive roles in the lives of their TGD relative. Additionally, the perceived parental support from parent 1 (most often the mother) and parent 2 (most often the father) was similar. Whilst other studies with mothers of TGD youth have reported that fathers seem slower to acceptance than mothers (Kuvalanka & Munroe, 2020), data (both quantitative and qualitative) were present in our study which showed that there are fathers who are just as, or more, gender affirming than mothers. Findings also suggested that parents needed to demonstrate a “readiness” for education to be effective in improving relations. Future research should look to further explore what factors contribute toward parental readiness to engage in education on gender diversity.

## Conclusion

While many TGD people in this study were able to address these narratives and educate their parents, this was a journey and something that took patience and perseverance. Even then, parental support was perceived as complex, confusing and inconsistent. Young people would often describe conditions to their gender transition that placed restrictions on their ability to live authentically. TGD youth (like other LGB youth) are vulnerable to family rejection since they usually are still living at home and are dependent on parents or guardians, so they may consider “support with strings” as adequate if it means they are able to remain at home. This may manifest as feeling themselves to be supported either directly or indirectly by their parents and may also result in a TGD young person having to manage relationships between parents with dissenting opinions. Parental support is clearly important for TGD youth for their psychological well-being and QoL, yet our findings indicate that there remains work to be done to improve parental support among these individuals. Nonetheless, we take hope from the many other examples of TGD youth living with support, solidarity, love and acceptance, with a parent/parent(s).

## Data Availability

The participants of this study did not give written consent for their data to be shared publicly, so due to the sensitive nature of the research supporting data is not available.
